# Association of nucleos(t)ide analogue treatment history with mortality in HBV-DNA-positive HBV-ACLF: a multicenter cohort study

**DOI:** 10.3389/fimmu.2026.1942114

**Published:** 2026-09-23

**Authors:** Liping Shen, Zhiqiang Jin, Fang Wei, Jie Li, Sijing Li, Xiaohao Wang, Hu Li, Shan Zhong

**Affiliations:** 1 Department of Infectious Diseases, Key Laboratory of Molecular Biology for Infectious Diseases (Ministry of Education), Institute for Viral Hepatitis, the Second Affiliated Hospital, Chongqing Medical University, Chongqing, China; 2 Department of Hepatology, Chongqing University Three Gorges Hospital, Chongqing, China

**Keywords:** acute-on-chronic liver failure, antiviral resistance, HBV-DNA, hepatitis B virus, mortality, nucleos(t)ide analogue, treatment withdrawal

## Abstract

**Background and aims:**

Hepatitis B virus-related acute-on-chronic liver failure (HBV-ACLF) is associated with high short- and long-term mortality. Prior nucleos(t)ide analogue (NA) treatment history, particularly treatment withdrawal and virological resistance, may contribute to ACLF development and influence prognosis after onset. However, the clinical impact of different NA treatment histories in patients with HBV-ACLF remains insufficiently characterized. This study evaluated the associations of baseline HBV-DNA status and prior NA treatment history with mortality in patients with HBV-ACLF.

**Methods:**

This multicenter retrospective cohort study included hospitalized patients with HBV-ACLF from three tertiary hospitals in Chongqing, China, between January 2018 and December 2024. Patients were stratified according to baseline HBV-DNA status. Among HBV-DNA-positive patients, prior NA treatment history was categorized as NA naïve, NA unsupervised withdrawal, or NA suboptimal response(NA-SR). Cox proportional hazards models were used to evaluate associations with 28-, 90-, 180-, and 360-day mortality. Propensity score matching was applied to reduce baseline confounding. Because of the small size of the NA-SR group, these analyses were considered exploratory.

**Results:**

Among 702 patients with HBV-ACLF, 625 were HBV-DNA-positive and 77 were HBV-DNA-negative at admission. Baseline HBV-DNA positivity showde a consistend trendtoward increased 28-day mortality (HR 2.19, 95% CI 0.83-5.83; P = 0.115). Among HBV-DNA-positive patients, compared with NA-naïve patients (n=480; 136 events), prior NA unsupervised withdrawal (n=127; 47 events) was associated with increased 360-day mortality (adjusted HR 1.41, 95% CI 1.00-2.00; P = 0.051), while NA suboptimal response (n=18; 11 events) showed a higher point estimate for 360-day mortality (adjusted HR 2.68, 95% CI 1.43-5.03; P = 0.002). Propensity score-matched analyses yielded directionally consistent results.

**Conclusion:**

Among HBV-DNA-positive patients with HBV-ACLF, prior NA unsupervised withdrawal showde a borderline associated with worse long-term survival, and NA suboptimal response showed a stronger and statistically significant association with worse long-term survival that requires confirmation in larger cohorts. These findings highlight NA treatment history as a clinically relevant disease-background factor in HBV-ACLF and support careful documentation of antiviral exposure, avoidance of unsupervised treatment discontinuation, and timely monitoring for virological breakthrough in chronic HBV care.

## 1 Introduction

Acute-on-chronic liver failure (ACLF) is a severe clinical syndrome characterized by acute hepatic decompensation, severe systemic inflammation, multiple organ failure, and high short-term mortality (1, 2). In the Asia-Pacific region, Hepatitis B virus (HBV) infection remains the predominant cause of ACLF (3). Despite improvements in intensive care, antiviral therapy, artificial liver support, and liver transplantation, the prognosis of HBV-related ACLF (HBV-ACLF) remains poor, particularly in patients without timely access to transplantation (4, 5).

Nucleos(t)ide analogues (NAs) are the cornerstone of chronic hepatitis B (CHB) treatment and effectively suppress HBV replication, reduce hepatic decompensation, and lower the risk of cirrhosis progression and hepatocellular carcinoma (6–8). Current guidelines from APASL (2), EASL (9), and AASLD (10) differ substantially regarding nucleos(t)ide analogue discontinuation: APASL and EASL permit finite therapy in selected non-cirrhotic patients under close monitoring, whereas AASLD recommends against withdrawal until HBsAg loss. However, in real-world practice, HBV-ACLF still occurs in patients with different prior antiviral treatment histories. These include NA-naïve patients, patients who discontinue NA therapy without physician supervision or without meeting guideline-recommended stopping criteria, and patients with persistent or breakthrough viral replication during NA therapy, suggesting antiviral suboptimal virological control (11–14).

Previous studies have shown that NA unsupervised withdrawal can trigger severe hepatitis flares, hepatic decompensation, and poor transplant-free survival in patients with chronic HBV infection (15–18). Mechanistically, NA suboptimal response may reflect the selection of drug-resistant HBV quasispecies under continuous drug pressure, with resistance-associated polymerase mutations (e.g., rtM204V/I, rtL180M, rtA181T/V, rtN236T, rtM250V) causing virological breakthrough and conferring cross-resistance that limits rescue options (11, 12). Clinically, this may present as prolonged ineffective viral suppression, delayed treatment adjustment, and insufficient monitoring of virological breakthrough, all of which may worsen liver injury and subsequent outcomes (12, 13). Because genotypic resistance testing is not universally available, persistent viremia may also represent low-level viremia or unrecognized non-adherence rather than confirmed resistance (13). However, the clinical significance of different prior NA treatment histories in patients who have already developed HBV-ACLF remains incompletely understood, especially in large real-world cohorts with extended follow-up.

Therefore, we conducted a multicenter retrospective cohort study to evaluate the associations of baseline HBV-DNA status and prior NA treatment history with short- and long-term mortality in patients with HBV-ACLF. Among HBV-DNA-positive patients, we further compared outcomes among NA-naïve patients, patients with prior NA unsupervised withdrawal, and patients with NA suboptimal response, in order to clarify the prognostic implications of these distinct antiviral treatment backgrounds. Although previous studies have examined the phenomenon of NA withdrawal in patients with HBV-ACLF (15, 18), this study introduces a new subgroup of patients with NA suboptimal response and includes an extended follow-up period of up to 360 days in a large, multicentre cohort.

## 2 Methods

### 2.1 Study design and participants

This multicenter retrospective cohort study included hospitalized patients with HBV-ACLF from three tertiary hospitals in Chongqing, China: The Second Affiliated Hospital of Chongqing Medical University, Chongqing University Three Gorges Hospital, and The Dazu Affiliated Hospital of Chongqing Medical University between January 2018 and December 2024. HBV-ACLF was diagnosed according to the APASL diagnostic criteria, The APASL diagnostic criteria for HBV-related ACLF include jaundice (serum total bilirubin >=5 mg/dL) and coagulopathy (international normalized ratio [INR] >= 1.5 or prothrombin activity <= 40%), with ascites and/or encephalopathy developing within 4 weeks in patients with preexisting chronic HBV infection associated liver disease (2).

Exclusion criteria were: (1) age < 18 years; (2) extrahepatic organ failure unrelated to ACLF; (3) active malignancy; (4) co-infection with other hepatitis viruses or human immunodeficiency virus (HIV); (5) prior liver transplantation; (6) use of immunosuppressants within 6 months before enrollment; (7) unclear history of antiviral therapy; (8) length of hospital stay: 7 days or less and (9) unavailable baseline HBV-DNA result.

To ensure data quality and consistency across centers, a standardized data harmonization protocol was used. Data extractors received centralized training, variable definitions were standardized, and random samples were cross-checked by principal investigators. Discrepancies were resolved by consensus. The study protocol was approved by the ethics committees of all participating institutions: the Second Affiliated Hospital of Chongqing Medical University [Approval No: 2025(307)], Chongqing University Three Gorges Hospital [Approval No: 2024(122)], and The Dazu Affiliated Hospital of Chongqing Medical University [Approval No: 2026(149)]. The requirement for informed consent was waived because of the retrospective design and the use of de-identified data. The study was conducted in accordance with the Declaration of Helsinki and reported according to the STROBE statement (**Supplementary Table 1**). The number of patients enrolled from each center is summarized in **Supplementary Table 2**.

### 2.2 Baseline HBV-DNA status and NA treatment history

Baseline HBV-DNA was measured at admission or within the first 48 hours of hospitalization. HBV-DNA-positivity was defined as detectable HBV-DNA above the validated lower limit of detection (20 IU/mL) used by each participating laboratory. HBV-DNA levels were log10-transformed for analysis. Among HBV-DNA-positive patients, prior NA treatment history was categorized into three groups.

#### 2.2.1 NA naïve group

Patients with no prior exposure to NAs or other anti-HBV antiviral drugs before admission.

#### 2.2.2 NA unsupervisded withdrawal group

Patients who had previously received NA therapy but discontinued treatment for more than 3 months before admission without physician supervision. Withdrawal history was verified using medical records, patient or family interviews, and, when available, prescription or pharmacy records. Detailed data on the duration of discontinuation, HBeAg status at stopping, prior treatment duration, and reasons for discontinuation were not consistently available and are therefore not included in the primary analysis.

#### 2.2.3 NA suboptimal response group (NA-SR)

Patients with persistent or breakthrough HBV-DNA positivity after at least 6 months of continuous NA therapy before admission, in the absence of documented poor adherence. The 6-month evaluation window is consistent with current EASL and AASLD guidance on the assessment of virological response to nucleos(t)ide analogue therapy (9, 10). NA suboptimal response was clinically adjudicated by hepatologists based on virological breakthrough, duration and type of prior NA therapy, prescription records, and patient or family interviews suggesting continued medication use. Cases with clear evidence of poor adherence were not classified as NA suboptimal response.

### 2.3 Clinical management protocol

All patients received standard-of-care management for HBV-ACLF, including nutritional support and complication-directed therapies. Antiviral therapy was evaluated and administered according to contemporary clinical guidelines, At our institution, entecavir and tenofovir are the standard first-line medications. The management of specific complications (e.g., ascites, infection, gastrointestinal bleeding, hepatic encephalopathy) followed established institutional protocols.

### 2.4 Outcomes

The primary outcomes were all-cause mortality at 28, 90, 180, and 360 days after admission. Because HBV-ACLF is characterized by both high short-term mortality and sustained long-term risk among survivors, all four time points were prespecified as co-primary endpoints. Survival status was ascertained from electronic medical records. For patients discharged with unknown vital status, trained research assistants conducted telephone follow-up with patients or immediate family members. The follow-up cutoff date was 30 June 2025.

During the follow-up period, 3 patients underwent liver transplantation (NA naïve:1; NA unsupervised withdrawal: 1; NA-SR: 1). Because of the small number of transplantation, no formal competing-risk analysis was performed; these patients were censored at the date of transplantation in the primary analysis.

Clinical complications, including infection, gastrointestinal bleeding, hepatic encephalopathy, Fungi, and ascites, were initially assessed during routine care and subsequently re-adjudicated according to predefined diagnostic criteria by two independent hepatologists. Disagreements were resolved by a senior hepatologist.

### 2.5 Statistical analysis

Statistical analyses were performed using R version 4.4.2 and Python version 3.12. Continuous variables are presented as median with interquartile range and categorical variables as number with percentage. Between-group comparisons were performed using the Mann–Whitney U test or Kruskal–Wallis test for continuous variables and the chi-square test or Fisher’s exact test for categorical variables, as appropriate. Because the NA-SR subgroup contained only 18 patients (11 deaths), multivariable models and propensity score matching for this subgroup were considered exploratory.

Survival distributions were estimated using Kaplan–Meier curves and compared using the log-rank test. Cox proportional hazards models were used to estimate hazard ratios (HR) and 95% confidence intervals (CI) for 28-, 90-, 180-, and 360-day mortality. Multivariable models were adjusted for clinically relevant baseline variables, including demographic characteristics, laboratory parameters, HBV-DNA level, and complications. Follow-up time was calculated from admission to death or the last known follow-up date, with a data cutoff of June 30, 2025. Patients who had not reached 360 days of observation by the cutoff date were administratively censored.

For comparisons of NA treatment history among HBV-DNA-positive patients, NA naïve status was used as the reference group. Sequential multivariable Cox models were constructed:

Model 1 was unadjusted;

Model 2 adjusted for age, sex, height, and weight;

Model 3 additionally adjusted for alanine aminotransferase, total bilirubin, C-reactive protein, white blood cell count, blood urea nitrogen, creatinine, lactate, fibrin degradation products, international normalized ratio, prothrombin time, and HBV-DNA level;

Model 4 further adjusted for cirrhosis, infection, gastrointestinal bleeding, hepatic encephalopathy, Fungi, and ascites.

The fully adjusted Cox model was considered the primary analysis for the HBV-DNA–mortality association, and propensity score matching was performed as a sensitivity analysis.

Missing baseline covariates were handled using a prespecified single-imputation strategy. Exposure-defining variables, follow-up times and outcome indicators were not imputed. Numeric variables were imputed using bagged tree models with 50 trees, whereas categorical variables were imputed using the most frequent category. The proportion of missing data for each baseline variable is shown in **Supplementary Table 3**. Complete-case analyses were performed as sensitivity analyses to assess the robustness of the main findings (**Supplementary Table 4**). A two-sided P value <0.05 was considered statistically significant.

### 2.6 Role of the funding source

The funding sources had no role in study design, data collection, data analysis, data interpretation, manuscript preparation, or the decision to submit the manuscript.

## 3 Results

### 3.1 Patient selection and baseline characteristics

A total of 1492 hospitalized patients with suspected HBV-ACLF were screened between January 2018 and December 2024. After applying exclusion criteria, 702 patients were included in the final cohort. Among them, 625 patients were HBV-DNA-positive at admission and 77 patients were HBV-DNA-negative. Among the HBV-DNA-positive patients, 18 had NA-SR, 127 had prior NA unsupervised withdrawal, and 480 were NA naïve. The patient selection flowchart is shown in **Figure 1**.

**Figure 1:**
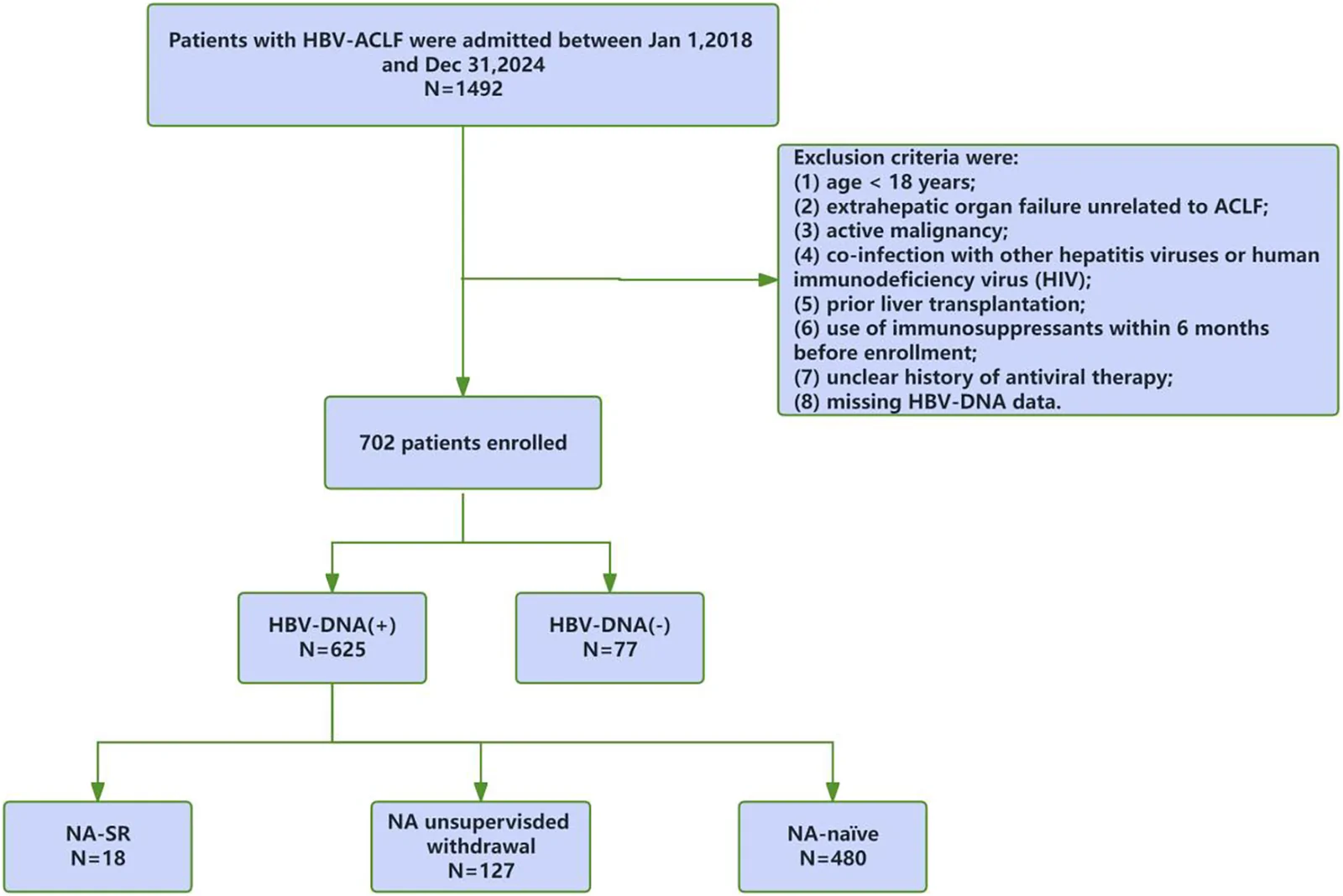
Flowchart of the screening process for the ACLF cohort. Flowchart of patients screened and included in the multicenter hepatitis B virus-related acute-on-chronic liver failure cohort. Among 1, 492 screened patients, 702 were included in the final analysis. Patients were first stratified according to baseline HBV-DNA status and then, among HBV-DNA-positive patients, according to prior nucleos(t)ide analogue treatment history.

Baseline characteristics of HBV-DNA-positive patients according to NA treatment history are shown in **Table 1**. Patients with NA-SR were older than NA naïve patients and had a higher prevalence of liver cirrhosis, as well as higher CRP and FDP levels. However, there was no statistically significant difference in the MELD, COSSH scores among the three groups. Follow-up was complete through 360 days for the majority of patients, and 28 patients were administratively censored before 360 days because of the fixed follow-up cutoff.

**Table 1:** Baseline characteristics of HBV-DNA(+) ACLF patients stratified by nucleos(t)ide analogue (NA) treatment pattern.

	NA naïve N = 480^1^	NA unsupervisded withdrawal N = 127^1^	NA-SR N = 18^1^	p-value^2^
Age(year)	51 (41, 58)	48 (42, 56)	56 (48, 64)	0.076
Male, n(%)	387 (81%)	109 (86%)	14 (78%)	0.319
Height(cm)	167 (161, 169)	167 (160, 168)	165 (160, 168)	0.452
Weight(kg)	65 (60, 72)	66 (60, 73)	65 (55, 70)	0.627
BMI(kg/m^2)^	23.9 (21.9, 26.1)	24.2 (21.8, 26.2)	23.4 (21.1, 26.9)	0.705
ALT(U/L)	429 (167, 903)	336 (159, 907)	288 (85, 876)	0.334
AST(U/L)	314 (142, 725)	311 (151, 606)	360 (131, 672)	0.976
GGT(U/L)	100 (67, 146)	107 (61, 158)	103 (73, 127)	0.804
TBIL(μmol/L)	255 (181, 334)	283 (194, 393)	290 (228, 354)	0.095
DBIL(μmol/L)	186 (126, 247)	205 (139, 279)	207 (161, 285)	0.066
Albumin(g/L)	31.2 (28.4, 34.3)	31.0 (28.0, 34.3)	30.0 (27.2, 32.0)	0.321
ALP(IU/L)	145 (121, 190)	143 (121, 196)	166 (121, 214)	0.838
Hb(g/L)	128 (114, 139)	127 (109, 139)	122 (105, 136)	0.437
WBC(10^9^/L)	6.5 (4.8, 9.1)	6.1 (4.5, 8.5)	5.8 (3.6, 7.4)	0.058
PLT(10^9^/L)	99 (72, 135)	83 (60, 123)	62 (49, 119)	0.005
CRP(mg/L)	13 (8, 20)	11 (7, 20)	20 (11, 34)	0.043
Ammonia(μmol/L)	50 (37, 65)	45 (33, 66)	53 (43, 73)	0.521
BUN(mmol/L)	4.70 (3.53, 6.36)	4.95 (3.42, 6.48)	5.21 (3.02, 8.00)	0.894
LAC(mmol/L)	3.03 (2.50, 3.74)	3.14 (2.63, 3.99)	3.44 (2.60, 3.91)	0.524
Cr(μmol/L)	62 (54, 76)	62 (54, 72)	55 (38, 71)	0.124
Na(mmol/L)	135.2 (132.6, 137.8)	135.4 (133.1, 137.5)	134.0 (131.5, 137.7)	0.554
K(mmol/L)	3.82 (3.47, 4.18)	3.72 (3.50, 4.17)	3.44 (3.27, 4.20)	0.187
FDP(mg/L)	7 (4, 11)	6 (3, 9)	9 (4, 17)	0.150
INR	2.04 (1.73, 2.48)	2.11 (1.73, 2.58)	1.93 (1.70, 2.40)	0.659
PT(s)	23.0 (20.1, 27.2)	23.8 (20.3, 28.2)	22.1 (19.8, 26.3)	0.508
D-dimer(mg/L)	618 (4, 1, 628)	511 (2, 1, 520)	582 (9, 1, 984)	0.534
Liver cirrhosis, n(%)	346 (72%)	112 (88%)	14 (78%)	<0.001
Infection, n(%)	408 (85%)	119 (94%)	17 (94%)	0.018
Gastrointestinal bleeding, n(%)	32 (6.7%)	9 (7.1%)	1 (5.6%)	0.949
Fungi, n(%)	79 (16%)	25 (20%)	2 (11%)	0.611
HBV-DNA(Log10 IU/ml)	5.81 (4.32, 7.00)	5.79 (4.08, 7.16)	5.54 (4.06, 6.50)	0.741
MELD Score	24.9 (22.8, 27.8)	24.9 (23.2, 28.8)	24.8 (22.7, 26.9)	0.433
MELD-Na Score	26.3 (23.7, 29.1)	26.5 (24.0, 29.7)	26.4 (24.4, 29.0)	0.571
COSSH Score	8.24 (7.74, 8.77)	8.26 (7.85, 8.76)	8.33 (7.85, 8.73)	0.545

^1^ Median (Q1, Q3); n (%).

^2^ Kruskal-Wallis rank sum test; Fisher’s Exact Test for Count Data with simulated p-value (based on 2000 replicates).

Baseline demographics, complications, laboratory variables, and prognostic scores are summarized across NA-naïve, NA NA unsupervisded withdrawal, and NA-SR groups. Continuous variables are presented as median (IQR) and categorical variables as n (%). P values were calculated using Kruskal–Wallis tests for continuous variables and Fisher’s exact tests (or χ² tests where appropriate) for categorical variables.

NA, nucleos(t)ide analogue;NA-SR, NA suboptimal response; MELD, Model for End-stage Liver Disease; COSSH-ACLF, Chinese Group on the Study of Severe Hepatitis B-ACLF score.

### 3.2 Baseline HBV-DNA status and mortality

Baseline table (**Supplementary Table 5**) shows that HBV-DNA-positive patients had higher indicators of liver damage, including higher ALT, AST and HBV-DNA levels. In the primary adjusted analysis, baseline HBV-DNA positivity showed a non-significant trend toward increased 28-day mortality (HR 2.19, 95% CI 0.83–5.83; P = 0.115). The association was stronger in the PSM sensitivity analysis (HR 3.82, 95% CI 1.13–12.94; P = 0.032), but no statistically significant association was observed at 90, 180, or 360 days (**Supplementary Tables 6**, **7**).

### 3.3 Mortality and HR of different NA treatment history in HBV-DNA-positive patients

During follow-up, 194 deaths occurred among the 625 HBV-DNA-positive patients, corresponding to a 360-day mortality rate of 31.0%. Kaplan–Meier analysis showed significant differences in 360-day survival among the NA suboptimal response, NA unsupervised withdrawal, and naïve groups (log-rank P<0.001; **Figure 2**), The 360-day mortality was 61.1% (11/18) in patients with NA-SR, 37.0% in NA unsupervised withdrawal patients, and 28.3% (136/480) in NA naïve patients. The unadjusted HR was highest in the NA suboptimal response group at all four evaluated time points (**Supplementary Table 8**), with an HR of 2.82 (95% CI 1.52-5.21; P = 0.001) for 360-day mortality. Besides, NA unsupervised withdrawal showed an association with increased 360-day mortality (HR 1.42, 95% CI 1.02-1.98; P = 0.038) and with 90- and 180-day mortality in unadjusted analyses (**Supplementary Table 8**), while associations at earlier time points were not statistically significant. Because genotypic resistance testing was not required by the NA-SR definition, this group should not be interpreted as confirmed NA resistance. Because of the small subgroup size, the NA-SR analyses should be regarded as exploratory and hypothesis-generating.

**Figure 2:**
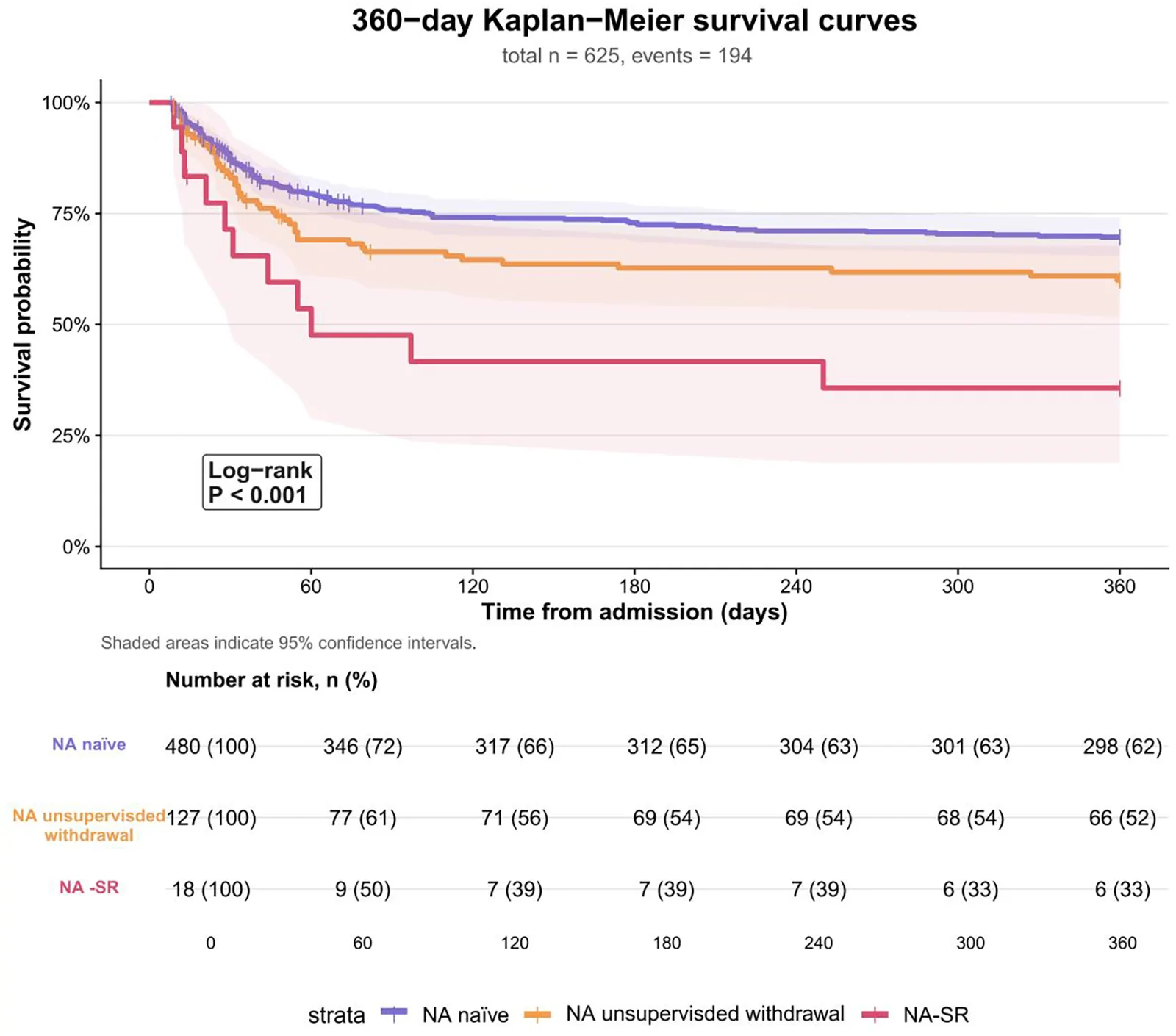
Kaplan–Meier survival curves according to nucleos(t)ide analogue treatment history. Kaplan–Meier curves for 360-day survival among HBV-DNA-positive patients stratified by prior NA treatment history: NA naïve, NA unsupervisded withdrawal, and NA suboptimal response. Survival differed significantly among groups by log-rank test. NA, nucleos(t)ide analogue; NA-SR, NA suboptimal response.

In multivariable Cox models for 360-day mortality, both NA unsupervised withdrawal and NA-SR remained associated with mortality after adjustment for demographics, laboratory parameters, HBV-DNA level, and complications, whereas the association for NA unsupervised withdrawal became borderline. Compared with NA naïve patients, the adjusted HR was 2.68 (95% CI: 1.43-5.03; P = 0.002) for NA-SR and 1.41 (95% CI: 1.00-2.00; P = 0.051) for NA unsupervised withdrawal (**Table 2**). This adjusted estimate is based on 11 events in the NA-SR subgroup and should not be interpreted as a stable measure of effect size.

**Table 2:** Sequential Cox models for 360-day mortality in HBV-DNA(+) patients.

	Model 1			Model 2			Model 3			Model 4		
Follow-up 360 days	HR	95% CI	p-value	HR	95% CI	p-value	HR	95% CI	p-value	HR	95% CI	p-value
NA naïve	Reference			Reference			Reference			Reference		
NA unsupervisde withdrawal	1.42	1.02, 1.98	0.038	1.47	1.06, 2.06	0.023	1.43	1.01, 2.01	0.041	1.41	1.00, 2.00	0.051
NA-SR	2.82	1.52, 5.21	<0.001	2.69	1.44, 5.01	0.002	2.83	1.50, 5.35	0.001	2.68	1.43, 5.03	0.002

CI, Confidence Interval, HR, Hazard Ratio.

Model 1: unadjusted.

Model 2: adjusted for age, sex, height, and weight.

Model 3: additionally adjusted for ALT, total bilirubin, C-reactive protein, white blood cell count, blood urea nitrogen, creatinine, lactate, fibrin degradation products, INR, prothrombin time, and HBV-DNA level.

Model 4: additionally adjusted for cirrhosis, infection, gastrointestinal bleeding, hepatic encephalopathy, Fungi, and ascites.

NA, nucleos(t)ide analogue; NA-SR, NA suboptimal response.

### 3.4 NA suboptimal response and NA unsupervised withdrawal as independent risk factors for mortality

In order to identify risk factors for survival in patients, univariate and multivariate Cox regression analyses were conducted. Data in **Table 3** indicated that some variables showed significant associations with mortality in univariate analysis. Following evaluation of univariate significance, clinical importance and multicollinearity (**Supplementary Table 9**), thirteen variables (Age, Direct bilirubin, Albumin, Creatinine, BUN, Lactate, CRP, FDP, INR, Cirrhosis, Infection, Bleeding and Fungi) were selected, along with NA suboptimal response and NA unsupervised withdrawal, to be included in the multivariate Cox regression model. After adjusting the confounders, NA unsupervised withdrawal (HR 1.389, 95% CI: 0.988-1.954; P = 0.059) showed a borderline association, whereas NA-SR (HR 2.561, 95% CI: 1.366-4.799; P = 0.003) was independently associated with increased 360-day mortality.

**Table 3:** Univariate and multivariate cox regression analyses for overall 360-day mortality of HBV-DNA(+) patients.

	Univariate analysis		Multivariate analysis	
Reference: NA naïve	HR (95% CI)	p	HR (95% CI)	p
**NA unsupervisde** **d withdrawal**	1.420 (1.019-1.979)	0.038	1.389 (0.988-1.954)	0.059
**NA** **-SR**	2.816 (1.523-5.208)	<0.001	2.561 (1.366-4.799)	0.003
Age (years)	1.036 (1.023-1.049)	<0.001	1.037 (1.023-1.050)	<0.001
Gender	0.997 (0.709-1.403)	0.986	–	
Height (cm)	0.987 (0.967-1.008)	0.223	–	
Weight (kg)	0.993 (0.981-1.005)	0.265	–	
HBV-DNA (Log10 IU/mL)	1.072 (0.986-1.164)	0.102	–	
ALT (U/L)	1.000 (1.000-1.000)	0.701	–	
AST (U/L)	1.000 (1.000-1.000)	0.213	–	
GGT (U/L)	0.999 (0.998-1.001)	0.368	–	
TBIL (μmol/L)	1.000 (1.000-1.000)	0.293	–	
DBIL (μmol/L)	1.004 (1.002-1.005)	<0.001	1.003 (1.001-1.004)	<0.001
Albumin (g/L)	0.976 (0.947-1.006)	0.117	1.008 (0.974-1.043)	0.658
Cr (μmol/L)	1.001 (1.000-1.003)	0.151	1.000 (0.997-1.003)	0.759
BUN (mmol/L)	1.025 (1.005-1.046)	0.012	1.002 (0.967-1.038)	0.905
LAC (mmol/L)	1.057 (0.973-1.149)	0.190	0.995 (0.900-1.101)	0.926
PLT (10^9^/L)	0.996 (0.993-0.999)	0.014	–	
WBC (10^9^/L)	1.025 (0.991-1.061)	0.154	–	
CRP (mg/L)	1.007 (1.003-1.011)	<0.001	1.005 (1.000-1.010)	0.073
FDP (mg/L)	1.010 (1.002-1.018)	0.015	1.003 (0.993-1.013)	0.525
D-dimer (mg/L)	1.000 (1.000-1.000)	0.008	–	
INR	1.350 (1.163-1.567)	<0.001	1.338 (1.106-1.619)	0.003
PT(s)	1.038 (1.019-1.057)	<0.001	–	
Liver cirrhosis	1.048 (0.755-1.456)	0.779	0.810 (0.572-1.146)	0.233
Infection	3.786 (1.939-7.392)	<0.001	2.841 (1.431-5.638)	0.003
Gastrointestinal bleeding	2.654 (1.701-4.141)	<0.001	2.062 (1.269-3.349)	0.003
Fungi	1.668 (1.191-2.335)	0.003	1.185 (0.830-1.692)	0.349
MELD score	1.087 (1.056-1.119)	<0.001	–	
MELD-Na score	1.094 (1.062-1.128)	<0.001	–	
COSSH score	2.131 (1.782-2.548)	<0.001	–	

CI, confidence interval; HR, hazard ratio. Endpoint: 360-day mortality.

NA, nucleos(t)ide analogue; NA-SR, NA suboptimal response.

Bold text indicates category headings, not statistical significance.

Compared with NA unsupervised withdrawal, NA-SR showed a consistently higher point estimate for 360-day mortality, among the baseline characteristics, Fungi, Direct bilirubin, Lactate, and INR were potentially the most important confounders in the relationship between NA-SR and all-cause mortality (**Table 4**).

**Table 4:** Relationship between NA-SR and 360-day mortality associated with each covariate in HBV-DNA(+) patients.

	NA-SR vs NA naïve			NA -SR vs NA unsupervisded withdrawal		
	HR	95% CI	p-value	HR	95% CI	p-value
**Unadjusted**	2.820	1.525-5.215	<0.001	1.966	1.019-3.794	0.044
**Adjusted**, adding ^a^						
Age	2.530	1.367-4.683	0.003	1.729	0.892-3.352	0.105
Liver cirrhosis	2.826	1.527-5.229	<0.001	1.968	1.016-3.812	0.045
Infection	2.613	1.412-4.835	0.002	1.964	1.018-3.789	0.044
Gastrointestinal bleeding	2.706	1.462-5.010	0.002	1.614	0.811-3.215	0.173
Fungi	2.909	1.571-5.386	<0.001	2.116	1.092-4.098	0.026
DBIL	2.653	1.434-4.908	0.002	2.006	1.039-3.873	0.038
Albumin	2.760	1.491-5.109	0.001	1.933	1.001-3.731	0.050
Cr	2.924	1.578-5.418	<0.001	1.970	1.012-3.833	0.046
BUN	2.764	1.494-5.113	0.001	1.948	1.009-3.762	0.047
LAC	2.817	1.523-5.211	<0.001	1.965	1.019-3.791	0.044
CRP	2.520	1.360-4.671	0.003	2.085	1.075-4.041	0.030
FDP	2.705	1.462-5.006	0.002	2.022	1.042-3.922	0.037
INR	3.016	1.626-5.592	<0.001	2.265	1.159-4.426	0.017

CI, Confidence Interval; HR, Hazard Ratio.

^a^ :Each of the covariates was added as a single confounder to a separate Cox proportional hazards model that contained only NA unsupervised withdrawal or NA suboptimal response. The adjusted HRs pertain to the effect of NA unsupervised withdrawal or NA suboptimal response on all-cause mortality after adjustment for that single covariate.

NA, nucleos(t)ide analogue; NA-SR, NA suboptimal response.

Bold text indicates category headings, not statistical significance.

### 3.5 PSM analyses for different NA treatment history

PSM was used to reduce baseline differences between groups (**Supplementary Tables 10**, **11**). Covariate balance before and after PSM was assessed using standardized mean differences (SMDs), the PSM procedure achieved acceptable covariate balance for both pairwise comparisons (**Supplementary Tables 12**, **13**). In matched analyses, the direction of association between NA treatment history and mortality remained consistent with the primary analysis. NA-SR versus NA unsupervised withdrawal remained significantly associated with 360-day mortality in both comparisons: versus NA unsupervised withdrawal (HR 4.479, 95% CI: 1.153-17.393; P = 0.030) and versus NA naïve (HR 3.118, 95% CI: 1.268-7.667; P = 0.013) (**Table 5**). In the unadjusted PSM sensitivity analyses (**Supplementary Table 14**), the comparison with NA unsupervised withdrawal remained significant (HR 2.134, 95% CI 1.009–4.517; P = 0.047), whereas the comparison with NA naïve did not reach statistical significance (HR 2.094, 95% CI 0.840–5.218; P = 0.113), likely reflecting the small size of the NA-SR subgroup. Overall, these analyses were directionally consistent with the primary findings.

**Table 5:** Multivariate cox regression analysis for overall 360-day mortality after PSM in HBV-DNA(+) patients.

	NA -SR vs NA naïve		NA-SR vs NA unsupervisded withdrawal	
After PSM	Multivariate HR (95% CI)	p	Multivariate HR (95% CI)	p
Overall	3.118 (1.268-7.667)	0.013	4.479 (1.153-17.393)	0.030
Age (years)	1.043 (1.016-1.071)	0.002	1.039 (1.016-1.063)	<0.001
Liver cirrhosis	0.539 (0.246-1.184)	0.124	0.792 (0.358-1.755)	0.566
Infection	2.473 (0.764-7.998)	0.131	0.942 (0.107-8.280)	0.957
Gastrointestinal Bleeding	1.136 (0.124-10.444)	0.910	28.437 (2.921-276.820)	0.004
Fungi	1.794 (0.775-4.151)	0.172	3.980 (1.083-14.633)	0.038
DBIL	1.005 (0.999-1.010)	0.084	0.997 (0.990-1.005)	0.500
Albumin	0.914 (0.822-1.017)	0.100	1.075 (0.916-1.263)	0.375
Cr	1.033 (1.006-1.060)	0.016	1.001 (0.979-1.023)	0.959
BUN	0.823 (0.710-0.954)	0.010	1.247 (1.046-1.487)	0.014
LAC	0.892 (0.664-1.198)	0.446	0.763 (0.530-1.098)	0.145
CRP	1.022 (0.999-1.045)	0.061	0.999 (0.974-1.025)	0.954
FDP	1.018 (1.012-1.025)	<0.001	1.455 (0.904-2.344)	0.123
INR	0.941 (0.404-2.196)	0.889	3.533 (1.492-8.362)	0.004

NA, nucleos(t)ide analogue; NA-SR, NA suboptimal response.

### 3.6 Sensitivity analyses

After additional adjustment for study center (**Supplementary Table 15**), the association between NA suboptimal response and 360-day mortality remained elevated (HR 2.55, 95% CI: 1.36-4.81; P = 0.004), and the association between NA unsupervised withdrawal and 360-day mortality was 1.37 (95% CI: 0.97-1.93; P = 0.071). Complete-case Cox analysis yielded directionally consistent results, with HRs of 1.52 (95% CI: 1.04-2.24; P = 0.031) for NA unsupervised withdrawal and 2.53 (95% CI: 1.21-5.29; P = 0.014) for NA suboptimal response.

## 4 Discussion

In this multicenter retrospective cohort study of patients with HBV-ACLF from three tertiary hospitals in Chongqing, baseline HBV-DNA positivity showed a consistent trend toward increased 28-day mortality, although the primary adjusted association did not reach statistical significance; this association was not observed at later time points. This temporal pattern suggests that active viral replication may play a particularly important role in the early phase of ACLF by contributing to acute immune-mediated hepatocyte injury and rapid hepatic deterioration (19–23). As the disease progresses, however, outcomes are likely to be increasingly influenced by hepatic reserve, cirrhosis burden, systemic inflammation, infection, and extrahepatic organ dysfunction rather than by the initial virological trigger alone (1, 24). These findings support the importance of prompt antiviral intervention in the acute stage, while also underscoring that longer-term survival depends on comprehensive management beyond viral suppression alone. Although the propensity-score-matched analysis showed a stronger association, the primary adjusted analysis did not reach statistical significance; therefore, the 28-day HBV-DNA finding should be interpreted as a trend rather than a confirmed effect.

A major finding of this study is that, among HBV-DNA-positive patients with HBV-ACLF, prior NA unsupervised withdrawal showed a borderline association with worse 360-day survival, and NA suboptimal response showed a stronger and statistically significant association with 360-day mortality. This observation nevertheless indicates that antiviral treatment history is not merely background information recorded before admission, but a clinically meaningful component of the disease course that may shape both the conditions preceding ACLF onset and the subsequent prognosis after ACLF has developed. In this context, NA treatment history should be regarded as an important element of clinical assessment in patients hospitalized with HBV-ACLF.

The association between prior NA unsupervised withdrawal and increased long-term mortality is consistent with previous evidence showing that inappropriate discontinuation of antiviral therapy may precipitate HBV reactivation, severe hepatitis flare, hepatic decompensation, and poor transplant-free survival (15–18). NA-SR was associated with the highest mortality in our cohort. Biologically, prolonged ineffective viral suppression may lead to persistent necroinflammation, cumulative liver injury, and further loss of hepatic reserve before ACLF develops, while delayed recognition of virological breakthrough or delayed adjustment to a more effective antiviral regimen may further worsen the clinical trajectory (11–13). Although the present retrospective design did not permit direct evaluation of time to rescue therapy, on-treatment HBV-DNA kinetics, cytokine profiles, or HBV-specific immune responses, these pathways are biologically plausible and should be investigated in future prospective studies. Notably, the adverse impact observed in the NA suboptimal response group suggests that this treatment history captures clinically relevant disease complexity that is not fully reflected by baseline severity measures alone; alternatively, residual confounding by unmeasured factors such as the specific NA agent, prior treatment duration, and post-admission antiviral management cannot be excluded. Notably, the adverse impact observed in the NA-SR group suggests that this treatment history captures clinically relevant disease complexity that is not fully reflected by baseline severity measures alone, alternatively, residual confounding by unmeasured factors such as the specific NA agent, prior treatment duration, and post-admission antiviral management cannot be excluded. From a practical standpoint, careful review of previous NA exposure, treatment response, virological breakthrough, and suspected resistance should be incorporated into routine evaluation at admission. The NA unsupervised withdrawal and NA suboptimal response categories represent heterogeneous and incompletely characterized exposures; limited data on the specific prior NA agent, treatment duration, duration off therapy, HBeAg status, EOT qHBsAg, and reasons for discontinuation restrict mechanistic interpretation of the observed associations. These mechanisms remain biologically plausible hypotheses that require direct evaluation in future prospective studies.

Beyond inpatient care, our findings also have implications for prevention in the outpatient setting. HBV-ACLF often represents the culmination of long-standing and potentially modifiable gaps in chronic HBV management, including poor adherence, NA unsupervised withdrawal, inadequate virological monitoring, and delayed recognition of treatment failure. A substantial body of evidence, synthesised in a recent scoping review, indicates that carefully selected HBeAg-negative, non-cirrhotic patients who stop NAs under structured off-treatment monitoring, particularly those with EOT qHBsAg <100 IU/mL, can achieve high sustained response rates and even functional cure, with hepatic decompensation rates below 1% (25). Our data largely reflect the unsupervised end of this spectrum—patients who stopped without qHBsAg-guided selection, cirrhosis exclusion, or scheduled ALT/HBV-DNA surveillance—rather than structured NA discontinuation per se. Strengthening long-term patient education, regular HBV-DNA surveillance, and timely optimization to potent agents with a high genetic barrier to resistance may help reduce the likelihood of severe acute deterioration and improve overall disease control.

Several limitations should be acknowledged. First, the retrospective observational design precludes causal inference, and residual confounding may persist despite multivariable adjustment and propensity score matching. Second, the number of patients in the NA-SR subgroup was small (n=18, 11 deaths). Given the limited sample size and the relatively large number of covariates, the multivariable models and propensity score matching had insufficient statistical power, and the associated effect estimates, have wide confidence intervals. These results should therefore be interpreted as exploratory and hypothesis-generating, and larger studies are needed to further clarify these findings. Third, genotypic resistance testing was not widely available in clinical practice. As a result, the NA-SR group, defined by persistent HBV-DNA positivity despite regular NA therapy for at least 6 months, may include patients with true antiviral resistance, suboptimal virological response, or unrecognized poor adherence. This may introduce some degree of classification uncertainty and could affect the precision of the effect estimates. Future studies with larger sample sizes and genotypic resistance testing are warranted to further refine the definition of NA-SR and validate these findings. Fourth, detailed data on the specific prior NA agent, duration of therapy, duration off therapy, HBeAg status and quantitative HBsAg at discontinuation, reasons for withdrawal, and quantitative adherence, serial HBV-DNA kinetics were not fully available, limiting mechanistic interpretation and adjustment for these potential confounders. Fifth, although this study included three centers, all are located within a single municipality (Chongqing) and predominantly use guideline-recommended first-line agents (entecavir, tenofovir disoproxil fumarate, and tenofovir alafenamide) in recent years. These findings may not generalize to settings where lamivudine or adefovir remain in widespread use, where the prevalence, patterns, and clinical consequences of NA resistance differ substantially. External validation in diverse geographic and healthcare settings is needed. Finally, Because patients hospitalized for 7 days or less were excluded to ensure complete baseline assessment, the present findings may not fully represent patients with very short hospital stays, including those who died or were transferred early; future studies with more complete early-phase data are warranted to address this concern.

In conclusion, among HBV-DNA-positive patients with HBV-ACLF, prior NA unsupervised withdrawal showed a borderline association with worse long-term survival, and NA suboptimal response showed a stronger signal toward worse long-term survival. These findings emphasize the importance of NA treatment history as a clinically relevant factor in the disease course of HBV-ACLF and support routine documentation of antiviral exposure, avoidance of unsupervised treatment discontinuation, and close monitoring for virological breakthrough in chronic HBV care.

## Data Availability

The data that support the findings of this study are available from the corresponding author on reasonable request.
