# Supplementary material for: Association of nucleos(t)ide analogue treatment history with mortality in HBV-DNA-positive HBV-ACLF: a multicenter cohort study
Source: Front Immunol. 2026 Sep 23;17:1942114. doi: 10.3389/fimmu.2026.1942114 (PMC13642767; doi:10.3389/fimmu.2026.1942114)
Supplement: Supplementary file 1 [file Table1.docx]

**Supplementary materials**

**Supplementary Table 1. STROBE statement**

**Supplementary Table 2. Patient enrolment by study center**

**Supplementary Table 3. Missingness of baseline variables**

**Supplementary Table 4. Complete-case Cox analysis**

**Supplementary Table 5. HBV-Status baseline**

**Supplementary Table 6. HBV-Status cox for four endpoints**

**Supplementary Table 7. Cox proportional hazards models across follow-up endpoints**

**Supplementary Table 8. Hazard Ratios for different nucleos(t)ide analogue treatment history in HBV-DNA(+) patients**

**Supplementary Table 9. Collinearity check**

**Supplementary Table 10. PSM baseline table resistance vs naive**

**Supplementary Table 11. PSM baseline table resistance vs withdrawal**

**Supplementary Table 12. PSM-SMD resistance vs naive**

**Supplementary Table 13. PSM-SMD resistance vs withdrawal**

**Supplementary Table 14. After PSM cox**

**Supplementary Table 15. Center-adjusted Cox analysis**

**Supplementary Table 1. STROBE statement—checklist of items that should be included in reports of cohort studies.**

|  | Page No. | Recommendation |
| --- | --- | --- |
| **Title and abstract** | 1-2 | (a) Indicate the study’s design with a commonly used term in the title or the abstract  (b) Provide in the abstract an informative and balanced summary of what was done and what was found |
| **Introduction** |  |  |
| Background/rationale | 4 | Explain the scientific background and rationale for the investigation being reported |
| Objectives | 4-5 | State specific objectives, including any prespecified hypotheses |
| **Methods** |  |  |
| Study design | 5 | Present key elements of study design early in the paper |
| Setting | 5 | Describe the setting, locations, and relevant dates, including periods of recruitment,  exposure, follow-up, and data collection |
| Participants | 5 | (a) Give the eligibility criteria, and the sources and methods of selection of participants. Describe methods of follow-up  (b) For matched studies, give matching criteria and number of exposed and unexposed |
| Variables | 5-6 | Clearly define all outcomes, exposures, predictors, potential confounders, and effect  modifiers. Give diagnostic criteria, if applicable |
| Data sources/ measurement | 6 | For each variable of interest, give sources of data and details of methods of assessment  (measurement). Describe comparability of assessment methods if there is more than  one group |
| Bias | 7 | Describe any efforts to address potential sources of bias |
| Study size | NA | Explain how the study size was arrived at |
| Quantitative variables | 7 | Explain how quantitative variables were handled in the analyses. If applicable, describe  which groupings were chosen and why |
| Statistical methods | 7 | (a) Describe all statistical methods, including those used to control for confounding  (b) Describe any methods used to examine subgroups and interactions  (c) Explain how missing data were addressed  (d) If applicable, explain how loss to follow-up was addressed  (e) Describe any sensitivity analyses |
| **Results** |  |  |
| Participants | 8 | (a) Report numbers of individuals at each stage of study—eg, numbers potentially  eligible, examined for eligibility, confirmed eligible, included in the study, completing  follow-up, and analysed  (b) Give reasons for non-participation at each stage  (c) Consider use of a flow diagram |
| Descriptive data | 8 | (a) Give characteristics of study participants (eg, demographic, clinical, social) and  information on exposures and potential confounders  (b) Indicate number of participants with missing data for each variable of interest  (c) Summarise follow-up time (eg, average and total amount) |
| Outcome data | 8 | Report numbers of outcome events or summary measures over time |
| Main results | 8-9 | (a) Give unadjusted estimates and, if applicable, confounder-adjusted estimates and their  precision (eg, 95% confidence interval). Make clear which confounders were adjusted  for and why they were included  (b) Report category boundaries when continuous variables were categorized  (c) If relevant, consider translating estimates of relative risk into absolute risk for a  meaningful time period |
| Other analyses | 9 | Report other analyses done—eg, analyses of subgroups and interactions, and sensitivity  analyses |
| **Discussion** |  |  |
| Key results | 10-11 | Summarise key results with reference to study objectives |
| Limitations | 11-12 | Discuss limitations of the study, taking into account sources of potential bias or  imprecision. Discuss both direction and magnitude of any potential bias |
| Interpretation | 12 | Give a cautious overall interpretation of results considering objectives, limitations,  multiplicity of analyses, results from similar studies, and other relevant evidence |
| Generalisability | 12 | Discuss the generalisability (external validity) of the study results |
| **Other information**  **Funding** | 12-14 | Give the source of funding and the role of the funders for the present study and, if applicable, for the original study on which the present article is based |

**Supplementary Table 2. Patient enrolment by study center.**

| Center | Screened (n) | Included (n) | Proportion (%) |
| --- | --- | --- | --- |
| The Second Affiliated Hospital of Chongqing Medical University(CQMU) | 1225 | 589 | 83.9 |
| Chongqing University Three Gorges Hospital(SX) | 161 | 52 | 7.4 |
| The Dazu Affiliated Hospital of Chongqing Medical University(DZ) | 106 | 61 | 8.7 |
| Total | 1492 | 702 | 100.00 |

Note: Data are presented as counts (n) and percentages. Percentages are calculated based on the total number of enrolled patients. The sum of proportions may not equal 100% due to rounding.

**Supplementary Table 3. Missingness of baseline variables.**

| **Variable** | **Missing After imputation** | **Missing Before imputation** | **Percent After imputation** | **Percent Before imputation** |
| --- | --- | --- | --- | --- |
| AARC_ACLF | 625 | 625 | 100 | 100.0 |
| ALB | 0 | 0 | 0 | 0.0 |
| ALP | 0 | 0 | 0 | 0.0 |
| ALT | 0 | 0 | 0 | 0.0 |
| AST | 0 | 0 | 0 | 0.0 |
| BUN | 0 | 1 | 0 | 0.2 |
| COSSH | 0 | 55 | 0 | 8.8 |
| CRP | 0 | 1 | 0 | 0.2 |
| Cr | 0 | 31 | 0 | 5.0 |
| DBIL | 0 | 1 | 0 | 0.2 |
| D-dimer | 0 | 0 | 0 | 0.0 |
| FDP | 0 | 101 | 0 | 16.2 |
| GGT | 0 | 78 | 0 | 12.5 |
| Hb | 0 | 0 | 0 | 0.0 |
| INR | 0 | 0 | 0 | 0.0 |
| K | 0 | 0 | 0 | 0.0 |
| L | 0 | 0 | 0 | 0.0 |
| LAC | 0 | 0 | 0 | 0.0 |
| MELD | 0 | 84 | 0 | 13.4 |
| MELD_Na | 0 | 1 | 0 | 0.2 |
| N | 0 | 2 | 0 | 0.3 |
| Ammonia | 0 | 0 | 0 | 0.0 |
| Na | 0 | 122 | 0 | 19.5 |
| PLT | 0 | 1 | 0 | 0.2 |
| PT | 0 | 0 | 0 | 0.0 |
| TBIL | 0 | 0 | 0 | 0.0 |
| WBC | 0 | 0 | 0 | 0.0 |
| AGE | 0 | 0 | 0 | 0.0 |
| art_liv | 0 | 0 | 0 | 0.0 |
| Ascites | 0 | 0 | 0 | 0.0 |
| Bleeding | 0 | 1 | 0 | 0.2 |
| BMI | 0 | 0 | 0 | 0.0 |
| Cirrhosis | 0 | 314 | 0 | 50.2 |
| Fungal | 0 | 0 | 0 | 0.0 |
| Hepatic Encephalopathy | 0 | 0 | 0 | 0.0 |
| High | 0 | 0 | 0 | 0.0 |
| Infection | 0 | 308 | 0 | 49.3 |
| lgHBV_DNA | 0 | 0 | 0 | 0.0 |
| Sex | 0 | 1 | 0 | 0.2 |
| Weight | 0 | 0 | 0 | 0.0 |

**Supplementary Table 4. Complete-case Cox analysis.**

| **Analysis** | **Characteristic** | **n** | **events** | **HR (95% CI)** | **p** |
| --- | --- | --- | --- | --- | --- |
| Primary imputed analysis | NA unsupervised withdrawal vs NA naïve | 625 | 194 | 1.389 (0.988-1.954) | 0.059 |
| Primary imputed analysis | NA-SR vs NA naïve | 625 | 194 | 2.561 (1.366-4.799) | 0.003 |
| Complete-case analysis | NA unsupervised withdrawal vs NA naïve | 494 | 154 | 1.524 (1.039-2.235) | 0.031 |
| Complete-case analysis | NA-SR vs NA naïve | 494 | 154 | 2.529 (1.208-5.294) | 0.014 |

Abbreviations: NA, nucleos(t)ide analogue;NA-SR,NA suboptimal response.

**Supplementary Table 5.HBV-Status baseline**

|  | **HBV-DNA(-)  N = 77^1^** | **HBV-DNA(+)  N = 625^1^** | **p-value^2^** |
| --- | --- | --- | --- |
| Age(year) | 52 (41, 56) | 50 (42, 58) | 0.983 |
| Male, n(%) | 64 (83%) | 510 (82%) |  |
| Height(cm) | 166.8 (163.0, 168.7) | 166.6 (160.0, 169.1) | 0.880 |
| Weight(kg) | 64 (58, 70) | 65 (60, 72) | 0.168 |
| BMI(kg/m2) | 23.7 (21.3, 25.4) | 24.0 (21.9, 26.1) | 0.098 |
| ALT(U/L) | 56 (38, 207) | 409 (163, 905) | <0.001 |
| AST(U/L) | 110 (65, 172) | 314 (144, 699) | <0.001 |
| GGT(U/L) | 60 (35, 98) | 102 (67, 149) | <0.001 |
| TBIL(μmol/L) | 290 (216, 406) | 263 (185, 346) | 0.080 |
| DBIL(μmol/L) | 211 (140, 310) | 190 (128, 256) | 0.057 |
| ALB(g/L) | 30.2 (27.1, 33.6) | 31.1 (28.3, 34.2) | 0.215 |
| ALP(IU/L) | 141 (107, 171) | 145 (121, 193) | 0.023 |
| Hb(g/L) | 116 (92, 130) | 127 (113, 139) | <0.001 |
| WBC(109/L) | 6.3 (4.4, 9.2) | 6.4 (4.8, 8.9) | 0.663 |
| PLT(109/L) | 71 (41, 117) | 97 (67, 132) | <0.001 |
| CRP(mg/L) | 13 (7, 27) | 13 (8, 20) | 0.569 |
| Ammonia(μmol/L) | 43 (29, 61) | 49 (36, 66) | 0.076 |
| BUN(mmol/L) | 5.1 (3.9, 7.6) | 4.8 (3.5, 6.4) | 0.035 |
| LAC(mmol/L) | 3.07 (2.34, 3.86) | 3.06 (2.52, 3.77) | 0.834 |
| Cr(μmol/L) | 65 (50, 82) | 62 (54, 75) | 0.399 |
| Na(mmol/L) | 135.2 (130.0, 138.0) | 135.2 (132.6, 137.8) | 0.460 |
| K(mmol/L) | 3.55 (3.33, 4.00) | 3.80 (3.46, 4.17) | 0.016 |
| FDP(mg/L) | 8 (4, 14) | 7 (4, 11) | 0.159 |
| INR | 1.98 (1.72, 2.53) | 2.05 (1.73, 2.50) | 0.585 |
| PT(s) | 22.6 (20.1, 27.4) | 23.0 (20.2, 27.4) | 0.706 |
| D-dimer(mg/L) | 255 (4, 1,517) | 593 (4, 1,580) | 0.489 |
| **Liver cirrhosis, n(%)** | 69 (90%) | 472 (76%) | 0.004 |
| **Infection, n(%)** | 67 (87%) | 544 (87%) | >0.999 |
| **Gastrointestinal Bleeding, n(%)** | 10 (13%) | 42 (6.7%) | 0.062 |
| **Fungal Infection, n(%)** | 9 (12%) | 106 (17%) | 0.326 |
| **HBV-DNA( Log10 IU/ml)** | 0.00 (0.00, 0.00) | 5.79 (4.31, 7.00) | <0.001 |
| **MELD Score** | 25.4 (22.6, 28.8) | 24.9 (22.9, 27.9) | 0.676 |
| **MELD-Na Score** | 27.3 (24.1, 30.4) | 26.4 (23.8, 29.3) | 0.232 |
| **COSSH Score** | 8.30 (7.82, 9.01) | 8.25 (7.78, 8.76) | 0.595 |
| ^1^Median (Q1, Q3); n (%) | | | |
| ^2^Kruskal-Wallis rank sum test; Fisher's exact test; Fisher's Exact Test for Count Data with simulated p-value (based on 2000 replicates) | | | |

Abbreviations: NA, nucleos(t)ide analogue;NA-SR,NA suboptimal response.

**Supplementary Table 6.HBV-Status cox for four endpoints**

| HBV-DNA(+) vs HBV-DNA(-) | Model 1 | | Model 2 | | Model 3 | | Model 4 | |
| --- | --- | --- | --- | --- | --- | --- | --- | --- |
| Endpoint | HR (95% CI) | p | HR (95% CI) | p | HR (95% CI) | p | HR (95% CI) | p |
| 28-day | 1.952 (0.790-4.824) | 0.148 | 1.902 (0.767-4.711) | 0.165 | 2.054 (0.792-5.326) | 0.139 | 2.194 (0.825-5.829) | 0.115 |
| 90-day | 1.122 (0.689-1.827) | 0.643 | 1.093 (0.670-1.783) | 0.722 | 1.071 (0.650-1.764) | 0.788 | 1.086 (0.653-1.807) | 0.751 |
| 180-day | 0.961 (0.622-1.484) | 0.857 | 0.938 (0.606-1.451) | 0.772 | 0.928 (0.595-1.450) | 0.744 | 0.928 (0.588-1.463) | 0.747 |
| 360-day | 0.887 (0.593-1.327) | 0.561 | 0.870 (0.580-1.304) | 0.500 | 0.856 (0.565-1.295) | 0.461 | 0.852 (0.558-1.301) | 0.458 |
| Abbreviations: CI = confidence interval; HR = hazard ratio. | | | | | | | | |
| Model 1: unadjusted. | | | | | | | | |
| Model 2: age + gender + high + weight. | | | | | | | | |
| Model 3: age + gender + high + weight + ALT + TBil + CRP + WBC + BUN + Cr + Lac + FDP + INR + PT. | | | | | | | | |
| Model 4: age + gender + high + weight + ALT + TBil + CRP + WBC + BUN + Cr + Lac + FDP + INR + PT + cirrhosis + infection + bleeding + hepatic encephalopathy + fungal infections + ascites. | | | | | | | | |

**Supplementary Table 7. Cox proportional hazards models across follow-up endpoints**

|  | **28-day** | | | **90-day** | | | **180-day** | | | **360-day** | | |
| --- | --- | --- | --- | --- | --- | --- | --- | --- | --- | --- | --- | --- |
| **Characteristic** | **HR** | **95% CI** | **p-value** | **HR** | **95% CI** | **p-value** | **HR** | **95% CI** | **p-value** | **HR** | **95% CI** | **p-value** |
| HBV-DNA(-) | Reference | | | Reference | | | Reference | | | Reference | | |
| HBV-DNA(+) | 3.82 | 1.13, 12.9 | 0.032 | 1.55 | 0.86, 2.80 | 0.145 | 1.46 | 0.85, 2.50 | 0.168 | 1.31 | 0.79, 2.18 | 0.290 |
| Abbreviations: CI = Confidence Interval, HR = Hazard Ratio | | | | | | | | | | | | |

**Supplementary Table 8. Hazard Ratios for different nucleos(t)ide analogue treatment history in HBV-DNA(+) patients**

|  | **28-day** | | | **90-day** | | | **180-day** | | | **360-day** | | |
| --- | --- | --- | --- | --- | --- | --- | --- | --- | --- | --- | --- | --- |
| **HBV-DNA(+)** | **HR** | **95% CI** | **p-value** | **HR** | **95% CI** | **p-value** | **HR** | **95% CI** | **p-value** | **HR** | **95% CI** | **p-value** |
| NA naïve | Reference | | | Reference | | | Reference | | | Reference | | |
| NA unsupervised withdrawal | 1.41 | 0.83, 2.38 | 0.202 | 1.47 | 1.02, 2.11 | 0.037 | 1.47 | 1.04, 2.08 | 0.029 | 1.42 | 1.02, 1.98 | 0.038 |
| NA-SR | 2.89 | 1.15, 7.23 | 0.023 | 2.70 | 1.37, 5.33 | 0.004 | 2.78 | 1.46, 5.29 | 0.002 | 2.82 | 1.52, 5.21 | <0.001 |
| Abbreviations: NA, nucleos(t)ide analogue;NA-SR,NA suboptimal response;CI = Confidence Interval, HR = Hazard Ratio | | | | | | | | | | | | |

**Supplementary Table 9. Collinearity check**

| **Variable 1** | **Variable 2** | **Spearman r** | **\|r\|** |
| --- | --- | --- | --- |
| INR | PT | 0.966 | 0.966 |
| DBIL | TBIL | 0.922 | 0.922 |
| MELD | MELD_Na | 0.921 | 0.921 |
| ALT | AST | 0.873 | 0.873 |
| INR | MELD | 0.804 | 0.804 |
| MELD | PT | 0.772 | 0.772 |
| INR | MELD_Na | 0.722 | 0.722 |
| Spearman correlation was used for preliminary collinearity screening. Pairs with \|r\| >= 0.7 should not generally be entered into the same multivariable model. | | | |

**Supplementary Table 10. PSM baseline table NA-SR vs naive**

|  | **HBV(+)-NA naive  N = 104^1^** | **HBV(+)-NA -SR  N = 17^1^** | **p-value^2^** |
| --- | --- | --- | --- |
| Age(year) | 54 (48, 60) | 55 (48, 60) | 0.662 |
| Male, n(%) | 87 (84%) | 13 (76%) | 0.493 |
| Height(cm) | 166 (161, 168) | 165 (160, 168) | 0.446 |
| Weight(kg) | 65 (59, 73) | 65 (56, 70) | 0.823 |
| BMI(kg/m2) | 23.9 (21.8, 26.1) | 24.5 (21.2, 26.9) | 0.955 |
| ALT(U/L) | 401 (185, 743) | 253 (85, 727) | 0.357 |
| AST(U/L) | 270 (139, 586) | 358 (131, 579) | 0.944 |
| GGT(U/L) | 99 (72, 145) | 102 (73, 120) | 0.864 |
| TBIL(μmol/L) | 276 (210, 349) | 288 (228, 339) | 0.991 |
| DBIL(μmol/L) | 204 (160, 261) | 201 (161, 267) | 0.958 |
| ALB(g/L) | 30.2 (27.5, 32.5) | 29.5 (27.2, 31.9) | 0.612 |
| ALP(IU/L) | 146 (122, 184) | 165 (121, 214) | 0.737 |
| Hb(g/L) | 126 (114, 136) | 123 (105, 136) | 0.558 |
| WBC(109/L) | 6.35 (4.58, 8.97) | 5.59 (3.61, 7.36) | 0.233 |
| PLT(109/L) | 92 (64, 121) | 63 (49, 119) | 0.447 |
| CRP(mg/L) | 15 (8, 20) | 17 (11, 28) | 0.112 |
| Ammonia(μmol/L) | 47 (33, 66) | 53 (44, 73) | 0.361 |
| BUN(mmol/L) | 4.96 (3.74, 6.51) | 5.01 (3.02, 6.83) | 0.709 |
| LAC(mmol/L) | 2.96 (2.48, 3.69) | 3.49 (2.60, 3.91) | 0.495 |
| Cr(μmol/L) | 56 (49, 64) | 53 (38, 67) | 0.553 |
| Na(mmol/L) | 134.3 (131.2, 136.8) | 134.0 (132.0, 137.7) | 0.785 |
| K(mmol/L) | 3.77 (3.43, 4.07) | 3.38 (3.27, 3.90) | 0.111 |
| FDP(mg/L) | 7 (4, 11) | 11 (4, 17) | 0.476 |
| INR | 1.97 (1.71, 2.42) | 1.94 (1.70, 2.40) | 0.914 |
| PT(s) | 22.6 (20.1, 26.5) | 22.6 (19.8, 26.3) | 0.777 |
| D-dimer(mg/L) | 1,040 (30, 2,026) | 676 (20, 1,984) | 0.840 |
| Liver cirrhosis, n(%) | 83 (80%) | 13 (76%) | 0.751 |
| Infection, n(%) | 102 (98%) | 16 (94%) | 0.368 |
| Gastrointestinal bleeding, n(%) | 2 (1.9%) | 1 (5.9%) | 0.368 |
| Fungal Infection, n(%) | 14 (13%) | 2 (12%) | >0.999 |
| HBV-DNA(Log10 IU/ml) | 5.06 (3.89, 6.76) | 5.61 (4.81, 6.50) | 0.709 |
| MELD Score | 24.95 (22.95, 26.95) | 24.40 (22.70, 26.00) | 0.644 |
| MELD-Na Score | 26.6 (24.1, 28.9) | 26.3 (24.4, 28.6) | 0.920 |
| COSSH Score | 8.47 (8.02, 8.85) | 8.28 (7.85, 8.64) | 0.586 |
| ^1^Median (Q1, Q3); n (%) | | | |
| ^2^Kruskal-Wallis rank sum test; Fisher's exact test; Fisher's Exact Test for Count Data with simulated p-value (based on 2000 replicates) | | | |

Abbreviations: NA, nucleos(t)ide analogue;NA-SR,NA suboptimal response.

**Supplementary Table 11. PSM baseline table NA-SR vs NA unsupervised withdrawal**

| **Characteristic** | **HBV(+)- NA unsupervised withdrawal  N = 63^1^** | **HBV(+)-NA -SR  N = 14^1^** | **p-value^2^** |
| --- | --- | --- | --- |
| Age(year) | 53 (44, 60) | 57 (51, 64) | 0.088 |
| Male, n(%) | 55 (87%) | 11 (79%) | 0.410 |
| Height(cm) | 166 (160, 169) | 166 (160, 168) | 0.696 |
| Weight(kg) | 66 (58, 77) | 66 (56, 70) | 0.561 |
| BMI(kg/m2) | 24.4 (21.8, 26.6) | 23.5 (21.1, 26.9) | 0.602 |
| ALT(U/L) | 308 (142, 844) | 339 (64, 876) | 0.890 |
| AST(U/L) | 306 (147, 571) | 400 (131, 672) | 0.756 |
| GGT(U/L) | 124 (61, 173) | 101 (67, 120) | 0.476 |
| TBIL(μmol/L) | 308 (215, 421) | 294 (228, 354) | 0.782 |
| DBIL(μmol/L) | 222 (161, 292) | 207 (176, 285) | 0.874 |
| ALB(g/L) | 29.9 (27.6, 32.8) | 29.3 (26.3, 31.9) | 0.366 |
| ALP(IU/L) | 145 (117, 191) | 135 (115, 195) | 0.668 |
| Hb(g/L) | 127 (112, 134) | 120 (103, 136) | 0.597 |
| WBC(10^9^/L) | 5.57 (3.75, 7.83) | 5.06 (3.61, 7.36) | 0.716 |
| PLT(10^9^/L) | 78 (52, 109) | 101 (49, 132) | 0.736 |
| CRP(mg/L) | 16 (7, 22) | 17 (10, 26) | 0.352 |
| Ammonia(μmol/L) | 49 (37, 70) | 53 (44, 85) | 0.480 |
| BUN(mmol/L) | 4.50 (3.22, 6.48) | 4.60 (3.02, 6.19) | 0.874 |
| LAC(mmol/L) | 3.14 (2.70, 3.79) | 3.19 (2.32, 3.81) | 0.653 |
| Cr(μmol/L) | 58 (52, 69) | 55 (31, 67) | 0.332 |
| Na(mmol/L) | 134.8 (132.0, 137.3) | 134.2 (132.1, 138.5) | 0.707 |
| K(mmol/L) | 3.70 (3.42, 4.17) | 3.38 (3.27, 4.20) | 0.202 |
| FDP(mg/L) | 6.2 (3.5, 10.3) | 6.5 (3.0, 14.3) | 0.575 |
| INR | 2.09 (1.74, 2.54) | 1.92 (1.63, 2.40) | 0.376 |
| PT(s) | 23.4 (20.4, 28.1) | 22.1 (19.6, 26.3) | 0.383 |
| D-dimer(mg/L) | 489 (2, 1,199) | 582 (9, 1,580) | 0.476 |
| Liver cirrhosis, n(%) | 52 (83%) | 10 (71%) | 0.455 |
| Infection, n(%) | 60 (95%) | 13 (93%) | 0.560 |
| Gastrointestinal bleeding, n(%) | 5 (7.9%) | 0 (0%) | 0.578 |
| Fungal Infection, n(%) | 11 (17%) | 2 (14%) | >0.999 |
| HBV-DNA(Log10 IU/ml) | 5.52 (3.74, 6.67) | 5.64 (4.81, 6.69) | 0.668 |
| MELD Score | 24.9 (23.3, 29.0) | 24.8 (22.2, 26.9) | 0.322 |
| MELD-Na Score | 26.6 (24.1, 29.9) | 26.3 (24.4, 28.6) | 0.472 |
| COSSH Score | 8.34 (8.01, 8.78) | 8.33 (7.89, 8.64) | 0.979 |
| ^1^Median (Q1, Q3); n (%) | | | |
| ^2^Kruskal-Wallis rank sum test; Fisher's exact test; Fisher's Exact Test for Count Data with simulated p-value (based on 2000 replicates) | | | |

Abbreviations: NA, nucleos(t)ide analogue;NA-SR,NA suboptimal response.

**Supplementary Table 12. PSM-SMD NA-SR vs naive**

|  | **Covariate** | **SMD before** | **SMD after** | **Balance after matching** |
| --- | --- | --- | --- | --- |
| NA-SR vs NA naïve | Lac | 0.035 | 0.256 | Imbalanced (>=0.2) |
|  | BUN | 0.103 | 0.235 | Imbalanced (>=0.2) |
|  | ALB | 0.397 | 0.233 | Imbalanced (>=0.2) |
|  | CRP | 0.373 | 0.189 | Acceptable (0.1-0.2) |
|  | COSSH | 0.312 | 0.130 | Acceptable (0.1-0.2) |
|  | high | 0.289 | 0.124 | Acceptable (0.1-0.2) |
|  | sex | 0.122 | 0.123 | Acceptable (0.1-0.2) |
|  | Cr | 0.888 | 0.106 | Acceptable (0.1-0.2) |
|  | FDP | 0.184 | 0.079 | Good (<0.1) |
|  | age | 0.526 | 0.079 | Good (<0.1) |
|  | cirrhosis | 0.057 | 0.078 | Good (<0.1) |
|  | weight | 0.096 | 0.075 | Good (<0.1) |
|  | lgHBV_DNA | 0.222 | 0.073 | Good (<0.1) |
|  | PT | 0.178 | 0.065 | Good (<0.1) |
|  | PLT | 0.513 | 0.059 | Good (<0.1) |
|  | INR | 0.132 | 0.051 | Good (<0.1) |
|  | MELD_Na | 0.022 | 0.049 | Good (<0.1) |
|  | infection | 0.094 | 0.045 | Good (<0.1) |
|  | bleeding | 0.011 | 0.044 | Good (<0.1) |
|  | WBC | 0.234 | 0.042 | Good (<0.1) |
|  | DBil | 0.283 | 0.015 | Good (<0.1) |
|  | fungal | 0.053 | 0.015 | Good (<0.1) |
|  | distance | 1.003 | 0.004 | Good (<0.1) |
|  | MELD | 0.168 | 0.002 | Good (<0.1) |

Abbreviations: NA, nucleos(t)ide analogue;NA-SR,NA suboptimal response.

**Supplementary Table 13. PSM-SMD NA-SR vs NA unsupervised withdrawal**

|  | **Covariate** | **SMD before** | **SMD after** | **Balance after matching** |
| --- | --- | --- | --- | --- |
| NA-SR vs NA unsupervised withdrawal | Lac | 0.046 | 0.429 | Imbalanced (>=0.2) |
|  | sex | 0.002 | 0.343 | Imbalanced (>=0.2) |
|  | CRP | 0.398 | 0.307 | Imbalanced (>=0.2) |
|  | age | 0.573 | 0.224 | Imbalanced (>=0.2) |
|  | FDP_log | 0.340 | 0.158 | Acceptable (0.1-0.2) |
|  | BUN | 0.102 | 0.149 | Acceptable (0.1-0.2) |
|  | high | 0.198 | 0.126 | Acceptable (0.1-0.2) |
|  | cirrhosis | 0.104 | 0.119 | Acceptable (0.1-0.2) |
|  | DBil | 0.120 | 0.110 | Acceptable (0.1-0.2) |
|  | ALB | 0.304 | 0.104 | Acceptable (0.1-0.2) |
|  | COSSH | 0.218 | 0.093 | Good (<0.1) |
|  | PLT | 0.279 | 0.088 | Good (<0.1) |
|  | lgHBV_DNA | 0.225 | 0.080 | Good (<0.1) |
|  | WBC | 0.014 | 0.080 | Good (<0.1) |
|  | bleeding | 0.015 | 0.064 | Good (<0.1) |
|  | Cr | 0.662 | 0.062 | Good (<0.1) |
|  | MELD | 0.292 | 0.049 | Good (<0.1) |
|  | infection | 0.007 | 0.019 | Good (<0.1) |
|  | distance | 1.204 | 0.018 | Good (<0.1) |
|  | PT | 0.304 | 0.014 | Good (<0.1) |
|  | INR | 0.231 | 0.006 | Good (<0.1) |
|  | weight | 0.137 | 0.004 | Good (<0.1) |
|  | MELD_Na | 0.075 | 0.003 | Good (<0.1) |
|  | fungal | 0.086 | 0.002 | Good (<0.1) |
|  | Lac | 0.046 | 0.429 | Imbalanced (>=0.2) |

Abbreviations: NA, nucleos(t)ide analogue;NA-SR,NA suboptimal response.

**Supplementary Table 14. After PSM cox**

|  | **NA-SR vs NA naïve** | | | **NA-SR vs NA unsupervised withdrawal** | | |
| --- | --- | --- | --- | --- | --- | --- |
| **After PSM** | **HR** | **95%CI** | **p, value** | **HR** | **95%CI** | **p, value** |
| 28-day | 2.194 | 0.479-10.054 | 0.312 | 3.577 | 0.862-14.846 | 0.079 |
| 90-day | 2.034 | 0.773-5.353 | 0.151 | 1.923 | 0.846-4.372 | 0.119 |
| 180-day | 1.903 | 0.730-4.965 | 0.188 | 2.164 | 0.963-4.866 | 0.062 |
| 360-day | 2.094 | 0.840-5.218 | 0.113 | 2.134 | 1.009-4.517 | 0.047 |
| Abbreviations:Abbreviations: NA, nucleos(t)ide analogue;NA-SR,NA suboptimal response;CI=ConfidenceInterval,HR=HazardRatio | | | | | | |

**Supplementary Table 15. Center-adjusted Cox analysis.**

| **Model** | **Characteristic** | **HR (95% CI)** | **p** |
| --- | --- | --- | --- |
| Center-adjusted Cox model | NA unsupervised withdrawal vs NA naïve | 1.371 (0.973-1.931) | 0.071 |
| Center-adjusted Cox model | NA-SR vs NA naïve | 2.554 (1.357-4.808) | 0.004 |
